# Cervical lymph node metastases in patients with differentiated thyroid cancer: A new (and more relevant) indication of active surveillance?

**DOI:** 10.20945/2359-4292-2023-0436

**Published:** 2024-05-07

**Authors:** Jose Miguel Dora, Rafael Selbach Scheffel

**Affiliations:** 1 Universidade Federal do Rio Grande do Sul Faculdade de Medicina Hospital de Clínicas de Porto Alegre Porto Alegre RS Brasil Unidade de Tireoide, Hospital de Clínicas de Porto Alegre, Faculdade de Medicina, Universidade Federal do Rio Grande do Sul, Porto Alegre, RS, Brasil; 2 Universidade Federal do Rio Grande do Sul Instituto de Ciências Básicas da Saúde Departamento de Farmacologia Porto Alegre RS Brasil Departamento de Farmacologia, Instituto de Ciências Básicas da Saúde, Universidade Federal do Rio Grande do Sul, Porto Alegre, RS, Brasil

The timeless saying "medicine is an ever-changing science" remains as true as ever. Within the realm of thyroid cancer, recent years have witnessed a significant accumulation of evidence that has ushered in remarkable transformations in the principles that guide management decisions. We are evolving to achieve care that aligns proportionality (administering treatments according to the tumor’s aggressiveness), enhancing value-based and integrating patient preferences to prioritize patient-centered outcomes.

Despite the generally positive outlook for patients with differentiated thyroid carcinoma (DTC), approximately 30% of individuals experience persistent or recurrent disease, frequently found in cervical lymph nodes (1). Traditionally, the primary approach to managing cervical disease in these patients has been surgical intervention, with some patients undergoing multiple procedures in pursuit of disease control or cure. Unfortunately, these objectives are not consistently met, and surgery carries the risk of potential complications such as hypoparathyroidism and voice disturbances and imposes significant emotional, time, and financial burdens (2,3). Nevertheless, the 2015 American Thyroid Association (ATA) Management Guidelines for Adult Patients with Thyroid Nodules and DTC strongly recommended therapeutic compartmental central or lateral neck dissection for patients with biopsy-proven persistent or recurrent disease in cervical lymph nodes based on specific size criteria. These criteria include a minimum size of ≥ 8 mm in the central compartment and ≥ 10 mm in the lateral compartment (4).

In this issue of AE&M, Solórzano and cols. provide insights into the conservative management (active surveillance, AS) of lymph node metastasis of DTC patients (5). The authors report a single-center cohort of 32 adult patients with papillary thyroid carcinoma (PTC) from Chile. All patients studied had confirmed (n = 18, 56%) or presumed (n=14, 44%) lymph node metastasis and underwent an AS protocol that involves examination, thyroglobulin, antithyroglobulin antibodies, and neck ultrasound every six months during the first year and after that at 6 to 12-month intervals. After a median follow-up of 4.3 years (range 0.6-14.1 years), only four patients (12.5%) showed locoregional disease progression: two underwent surgery, one was treated with external beam radiation, and the other was awaiting surgery. Importantly, none of the four patients with lymph node progression displayed complications related to tumor growth or treatment of the metastasis. These results are consistent with previous literature that showed a 20% to 24% rate of cervical lymph node enlargement, including two recent cohorts published by Argentinian and Brazilian researchers (6,7).

Nowadays, AS for primary thyroid tumors (specifically those with less than 1 cm in size) is a well-accepted strategy, although not applied in all healthcare scenarios (8). The efficacy and safety of this treatment strategy for low-risk PTC is already demonstrated in several countries, with the most extended experience from Japan (30 years) with more than 3,000 patients followed (9). This mounting evidence led to guidelines and societies (including the Brazilian Society of Endocrinology and Metabolism) recommending AS as the initial approach in selected patients with microPTC (10). Moreover, a systematic review of the cost-effectiveness of AS compared to immediate surgery found that most studies favored AS (11).

The construct of conservative management of primary DTC has been expanded for lymph node metastasis, and accumulating evidence suggests that AS is a feasible strategy for this condition, given that most lymph node metastases identified following total thyroidectomy do not typically exhibit noticeable growth. One should note that AS is a reasonable strategy for small-volume cervical disease that do not threaten noble structures. Additionally, it should be conducted by healthcare teams with resources and knowledge to implement AS protocols for patients that accept conservative management.

One significant limitation of using AS for primary thyroid tumors is that the majority of actual recommendations do not advocate cytological evaluation of thyroid nodules less than 1 cm in size. As a result, most patients eligible for AS should not have been diagnosed at all. On the other hand, most patients with lymph node metastases are today treated surgically and AS has proven to be a viable choice in this scenario. Since these patients already have a diagnosis of DTC, the use of AS in managing persistent cervical disease seems to be a more relevant indication compared to its use in primary tumors.

In summary, the present study contributes to the growing body of evidence supporting the consideration of AS as a viable choice for specific cases among patients with DTC who have suspected or confirmed cervical lymph node metastases. Nevertheless, a definitive determination regarding the appropriateness of AS will ultimately hinge on the findings of prospective studies that meticulously outline criteria for patient and tumor selection and lay out comprehensive protocols for tracking disease progression. These forthcoming trials will play a pivotal role in shaping evidence-based recommendations to guide the AS approach for DTC cervical lymph node metastases.
